# Association Between Traditional Herbal Diet and Nasopharyngeal Carcinoma Risk: A Prospective Cohort Study in Southern China

**DOI:** 10.3389/fonc.2021.715242

**Published:** 2021-10-21

**Authors:** Yun-Hong Lyu, Chu-Yang Lin, Shang-Hang Xie, Tong Li, Qing Liu, Wei Ling, Yu-Qiang Lu, Su-Mei Cao, Ai-Hua Lin

**Affiliations:** ^1^School of Public Health, Sun Yat-Sen University, Guangzhou, China; ^2^Department of Cancer Prevention Center, Sun Yat-Sen University Cancer Center, Guangzhou, China; ^3^Sihui Cancer Institute, Sihui, China; ^4^State Key Laboratory of Oncology in South China, Collaborative Innovation Center for Cancer Medicine and Guangdong Key Laboratory of Nasopharyngeal Carcinoma Diagnosis and Therapy, Sun Yat-Sen University Cancer Center, Guangzhou, China; ^5^Guangzhou Xinhua University, Guangzhou, China

**Keywords:** cohort study, Epstein-Barr virus, herbal diet, nasopharyngeal carcinoma, risk factor

## Abstract

**Introduction:**

Prospective evidence for herbal diet and nasopharyngeal carcinoma (NPC) development is absent. We therefore evaluated the associations of herbal soup and herbal tea with NPC in a prospective cohort study in southern China.

**Methods:**

Based on an NPC screening cohort established in 2008–2015, information on herbal diet consumption, potential confounding factors, and Epstein-Barr virus (EBV) antibody levels were collected from 10,179 individuals aged 30–69 years in Sihui city, southern China. Cox regression models were performed to examine herbal diet with NPC risk, and logistic regression models were used to examine herbal diet with EBV reactivation.

**Results:**

During a median of 7.54 years of follow-up, 69 participants developed NPC. Herbal soup consumption was associated with decreased NPC risk, with HRs of 0.31 (95% confidence interval (CI): 0.15–0.62) for the highest intake frequency and 0.29 (95% CI: 0.16–0.51) for a longer duration. However, herbal tea was not significantly associated. Moreover, we identified herbal soup was inversely associated with EBV seropositivity among all the participants at baseline, with the adjusted ORs being 0.78 (95% CI: 0.65–0.93) for immunoglobulin A antibodies against EBV capsid antigens (VCA-IgA) and 0.76 (95% CI: 0.64–0.91) for nuclear antigen 1 (EBNA1-IgA) in those with the highest frequency and 0.70 (95% CI: 0.59–0.84) for VCA-IgA and 0.64 (95% CI: 0.54–0.77) for EBNA1-IgA in those with the longer duration. Inverse associations were also observed in non-NPC individuals.

**Conclusions:**

With inhibition of EBV reactivation by plants, herbal soup could significantly decrease the risk of NPC in endemic areas.

## Introduction

Nasopharyngeal carcinoma (NPC) is rare in most parts of the world but prevalent in southern China. In 2020, 133,354 new cases of NPC were diagnosed worldwide, and approximately half of the cases are from China (1). Epstein-Barr virus (EBV) is a well-established risk factor for NPC in the endemic regions (2, 3). The virus preferentially establishes latent infection in memory B cells after infection and can be reactivated into lytic phase by endogenous and exogenous factors (4–6). Such virus reactivation, reflected by elevated levels of serological antibodies against multiple EBV antigens, has been suggested as a key step of NPC onset and development (7, 8). However, the contradiction between ubiquitous EBV infection and distinctive geographic distribution of NPC suggests the involvement of other environmental factors, especially traditional diet, in the etiology of NPC (9–12).

Herbal teas and herbal soups, two traditional dietary staples with multiple herbal medicines, have been widely consumed in local populations of southern China for several centuries (13–15). An association between herbal diet and NPC has been hypothesized, but the results are inconsistent. Several studies reported that the use of herbal medicines was associated with an elevated risk of NPC (16–18), whereas some reported an inverse association (19, 20). The reasons for the discrepancy might be that previous studies did not separate the herbs nature and function in diet. In fact, some herbs are traditionally used for treating certain diseases (21–23) and were not commonly used as diet components (24). Another possibility might be due to inherent limitations of case-control studies in the inference of reverse causality and recall bias (17). Detailed information on intake frequency and duration in diet collected in a large, prospective cohort study would facilitate the rigorous evaluation of herbal diet for NPC.

Another unsolved question is whether herbal plants in diet link to NPC through its ability to interact with Epstein-Barr virus (EBV) infection. Since extractions from some herbal medicines can affect EBV reactivation (25–29), this interaction is biologically plausible; however, no large-scale studies have been conducted to validate the hypothesis. Therefore, to better understand the causes and make prevention for this major public health problem in endemic areas, we performed a prospective study to evaluate the associations of herbal soup and herbal tea with NPC in southern China.

## Methods

### Study Population

This cohort study was conducted based on an NPC screening program which was launched from 2008. Detailed descriptions of the project design and characteristics of participants have been given previously (30, 31). In brief, local residents aged 30–69 years were recruited between 2008 and 2015 from seven towns in Sihui city, Guangdong province, China. Each participant was required to complete a written informed consent and a face-to-face questionnaire related to demographic and dietary characteristics. The Institutional Research Ethics Committee of Sun Yat-Sen University Cancer Center (SYSUCC) approved this study.

A total of 10,839 residents completed the baseline questionnaires. We excluded participants who had been diagnosed with NPC previously (*n* = 28), those with missing data (*n* = 461) or those whose ages were not accordant with the inclusion criteria (*n* = 143) at baseline. Thus, 10,207 participants were included in this cohort at baseline, and their blood samples were collected by trained staff to measure the levels of EBV antibodies [immunoglobulin A antibodies against EBV capsid antigens (VCA-IgA) and nuclear antigen 1 (EBNA1-IgA)] by using the commercial enzyme-linked immunosorbent assay (ELISA) kits (produced by EUROIMMUNAG, Lübeck, Germany and Zhongshan Bio-Tech Company, Zhongshan, China) at Sun Yat-Sen University Cancer Center (SYSUCC). The detailed procedure of the EBV serological test was described in our previous study (31). According to the standards of ELISA kits, the positive criteria were ≥0.7 for EBNA1-IgA and ≥0.8 for VCA-IgA.

### Data Collection

Trained interviewers collected information through face-to-face interviews using structured questionnaires at baseline. Participants were asked to report their frequency and duration of herbal soup and herbal tea consumption. Frequency of herbal soup or herbal tea consumption was categorized as less than monthly, monthly or more, or weekly or more. The consumption duration was divided into two groups: ≤5 or >5 years. Consumption of herbal soup or tea at least once a month was defined as ever consumption, else defined as never consumption. In the current study, we collected the information on consumption of herbal soups and teas including the ones homemade or bought in markets or shops, whereas we did not investigate the specific ingredients in these herbal diets. However, through our previous case-control study in Sihui, we found that plants frequently used in herbal soups include *Semen coicis*, *Bulbus lilii*, *Polygonatum odoratum*, *Fructus lycii*, etc, and the most prevalent herbs used in teas are *Chrysanthemum morifolium*, *Prunella asiatica*, *Flos lonicerae*, *Glycyrrhiza uralensis*, *Helicteres angustifolia*, etc. (19).

Other covariates obtained from the questionnaires included age (<50 or ≥50 years), sex (male or female), education level (<6 or ≥6 years), family history of NPC (no or yes), combined levels of EBV antibodies (both negative or any positive), smoking status (never smoker, former smoker, or current smoker), fresh fruit intake (less than daily or daily), fresh vegetable intake (less than daily or daily), and salted food intake (less than monthly or monthly or more).

### Follow-Up and Case Ascertainment

According to the predefined prediction formula combined with the two EBV antibodies mentioned above (32), the participants were divided into high-risk, medium-risk, or low-risk subgroups. The low-risk participants were retested for EBV antibodies every 4–5 years, while those in the medium-risk or high-risk subgroup were retested annually. Follow-up durations were calculated from the date of recruitment to the date of NPC diagnosis, date of loss to follow-up, date of death, or the study deadline of December 31, 2016, whichever occurred first. The information of new NPC cases was obtained through periodic checkups in this screening cohort and cancer registries at the Sihui Cancer Institute. NPC cases and deaths were further identified by annually reviewing the causes of death registration data provided by the local Centers for Disease Control (CDC) and the death rosters of village committees.

**Figure 1:**
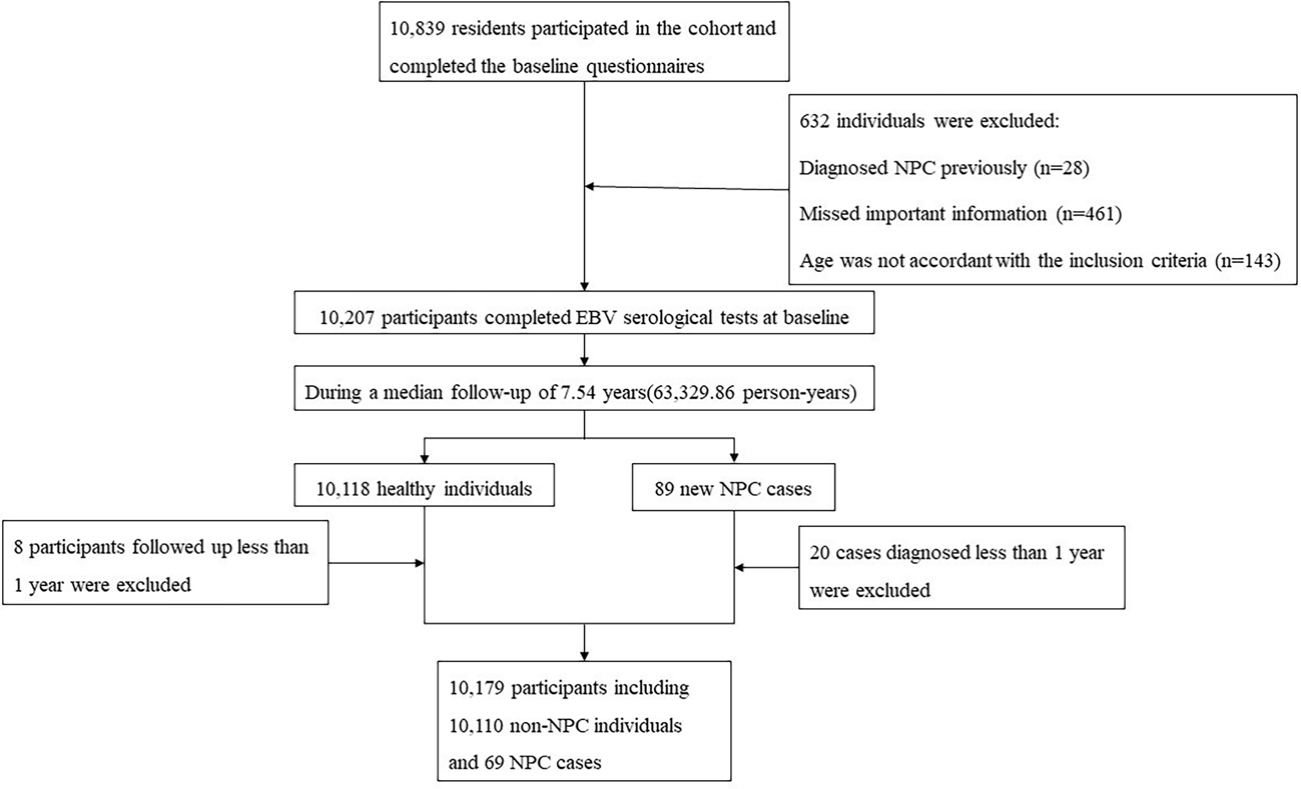
Flow chart of the recruitment process for participants.

During a median of 7.54 years of follow-up (63,329.86 person-years), 89 new NPC cases were identified. We excluded 20 cases who were diagnosed within 1 year after recruitment, as well as eight healthy participants who were followed-up less than 1 year. Eventually, 10,179 participants were available for statistical analysis, among which 69 were NPC cases diagnosed more than 1 year after recruitment: 46 from periodic checkups and 23 from cancer registries. The flow chart of the recruitment process for participants is shown in **Figure 1**.

### Statistical Analysis

NPC incidence rate was calculated by the number of new cases divided by the total person-years of follow-up. For the present analyses, age was divided into four categories (30–39, 40–49, 50–59, and 60–69 years). The combined EBV antibodies at baseline was defined as negative if both VCA-IgA and EBNA1-IgA were negative, else defined as positive if anyone of them was positive. Participants were grouped into categories based on intake frequency for herbal soup or herbal tea, and Chi-square tests were used to examine differences in baseline characteristics of participants among different groups.

Cox’s proportional hazard regression models were conducted to estimate the hazard ratios (HRs) and 95% confidence intervals (CIs) for the risk of NPC associated with herbal diet. In a minimally adjusted model, we adjusted for age and sex. The fully adjusted model additionally included education level, family history of NPC, combined levels of EBV antibodies, smoking status, salted food intake, and fresh vegetable and fresh fruit consumption. The proportional hazard (PH) assumption was checked for the main exposures (consumption of herbal soup and herbal tea), and no violation was found (*p* > 0.05). Linear trend tests were carried out by treating categorical variables as continuous variables. Combined associations of herbal diet intake frequency and duration with NPC were also evaluated with the lowest frequency and shorter duration as reference.

Stratified analyses were performed by the following variables: age, sex, education level, combined levels of EBV antibodies, smoking status, and salted food intake. The potential interactions were calculated by including multiplicative interaction terms in the Cox models, and likelihood ratio tests were used to examine statistical significance of these interaction terms.

In addition, logistic regression models were used to estimate odds ratios (ORs) and corresponding 95% CIs for the associations of herbal diet with VCA-IgA and EBNA1-IgA seropositivities. Due to the small number of NPC cases, we only performed the analyses among all the participants at baseline and healthy individuals in this cohort. The adjustments in models included sex, age, education level, family history of NPC, smoking status, salted food intake, and fresh vegetable and fresh fruit consumption.

All data were recorded using Epidata 3.1 by double entry. SAS software (version 9.4) and Stata software (version 16) were used for the analyses. All statistic tests were two sided with *α*-value at 0.05 considered to be statistically significant.

## Results

### Cohort Characteristics

Of 10,179 participants, 42.02% were men and 57.98% were women with a mean age of 48.74 ± 9.13 years. The overall incidence rate of NPC was 108.95 per 100,000 person-years, with 170.10 per 100,000 person-years for males and 65.09 per 100,000 person-years for females, respectively. Participants who consumed the two kinds of herbal diet in higher frequency were more likely to be younger and more educated. While participants who consumed herbal soup in higher frequency were more likely to be EBV seronegative, this trend was not found in those consuming herbal tea. Participants who consumed herbal tea in higher frequency were more likely to be male, to be current smokers, and to consume salted food more frequently. More baseline characteristics are presented in **Table 1**.

**Table 1:** Baseline characteristics of participants in a prospective cohort in southern China divided by frequency of herbal soup and herbal tea consumption (*N* = 10,179).

Variables	Herbal soup consumption^a^			*p-*value^b^	Herbal tea consumption^a^			*p-*value^b^
	Less than monthly	Monthly or more	Weekly or more		Less than monthly	Monthly or more	Weekly or more	
Participants (*n*, %)	1,070 (10.51)	4,140 (40.67)	4,969 (48.82)		5,036 (49.47)	3,667 (36.03)	1,476 (14.50)	
Sex								
Male	459 (42.90)	1,719 (41.52)	2,099 (42.24)	0.650	1,927 (38.26)	1,635 (44.59)	715 (48.44)	<0.001
Female	611 (57.10)	2,421 (58.48)	2,870 (57.76)		3,109 (61.74)	2,032 (58.41)	761 (51.56)	
Age (years)								
30–39	134 (12.52)	572 (13.82)	987 (19.86)	<0.001	683 (13.56)	719 (19.61)	291 (19.72)	<0.001
40–49	401 (37.48)	1,543 (37.27)	1,888 (39.00)		1,835 (36.44)	1,422 (38.78)	575 (38.95)	
50–59	372 (34.77)	1,408 (34.01)	1,510 (30.39)		1,666 (33.08)	1,153 (31.44)	471 (31.91)	
60–69	163 (15.23)	617 (14.90)	584 (11.75)		852 (16.92)	373 (10.17)	139 (9.42)	
Education year (years)								
<6	512 (47.85)	1,647 (39.78)	1,724 (34.70)	<0.001	2,115 (42.00)	1,224 (33.38)	544 (36.86)	<0.001
≥6	558 (52.15)	2,493 (60.22)	3,245 (65.30)		2,921 (58.00)	2,443 (66.62)	932 (63.14)	
Family history of NPC								
No	1,037 (96.92)	4,018 (97.05)	4,784 (96.28)	0.108	4,880 (49.60)	3,546 (96.70)	1,413 (95.73)	0.087
Yes	33 (3.08)	122 (2.95)	185 (3.72)		156 (45.90)	121 (3.30)	63 (4.27)	
Combined EBV antibodies^c^								
Both negative	524 (48.97)	2,105 (50.85)	2,646 (53.25)	0.010	2,568 (51.99)	1,945 (53.04)	762 (51.63)	0.166
Any positive	546 (51.03)	2,035 (49.15)	2,323 (43.75)		2,468 (49.01)	1,722 (46.96)	714 (48.37)	
Smoking status								
Never smoker	733 (68.50)	2,848 (68.79)	3,462 (69.67)	0.725	3,636 (72.20)	2,456 (66.98)	951 (64.43)	<0.001
Former smoker	48 (4.49)	189 (4.57)	238 (4.79)		206 (4.09)	187 (5.10)	82 (5.56)	
Current smoker	289 (27.01)	1,103 (26.64)	1,269 (25.54)		1,194 (23.71)	1,024 (27.92)	443 (30.01)	
Salted food intake								
Less than monthly	951 (88.88)	3,667 (88.57)	4,399 (88.53)	0.948	4,531 (89.97)	3,248 (88.57)	1,238 (83.88)	<0.001
Monthly or more	119 (11.12)	473 (11.43)	570 (11.47)		505 (10.03)	419 (11.43)	238 (16.12)	

a Values are presented as number (percentage).

b p-values were obtained using Chi-square tests.

c Combined EBV antibodies: the combined EBV antibodies were defined as negative if both VCA-IgA and EBNA1-IgA were negative, else defined as positive if anyone of them was positive. According to the standards of ELISA kits, the positive criteria were ≥0.7 for EBNA1-IgA and ≥0.8 for VCA-IgA.

### Herbal Diet and NPC Risk

**Table 2** shows the minimally and fully adjusted HRs of developing NPC associated with herbal tea or herbal soup. The association between herbal tea consumption and NPC was insignificant (fully adjusted HR: 1.32, 95% CI: 0.68–2.55). Also, we found neither frequency nor duration of herbal tea intake had a significant association with NPC risk, with fully adjusted HRs of 0.91 (95% CI: 0.35–2.32) for those consuming herbal tea weekly or more (*vs.* less than monthly) and 0.70 (95% CI: 0.37–1.34) for a duration of more than 5 years (*vs.* ≤5 years).

**Table 2:** Hazard ratios (HRs) and 95% confidence intervals (CIs) of developing nasopharyngeal carcinoma associated with herbal diet in a prospective cohort in southern China (*N* = 10,179).

Variables	Participants (*n* (%))	Person-years^a^	Nasopharyngeal carcinoma				
			Case (*n*)	Incidence rate	HR (95% CI)^b^	Fully adjusted HR (95% CI)^c^	*p*-value
Herbal soup intake							
Never consumption	1,070 (10.51)	7,026.34	23	327.34	1.00	1.00	
Ever consumption	9,109 (89.49)	56,303.52	46	81.70	0.27 (0.16, 0.45)	0.31 (0.16, 0.58)	0.001
Frequency of herbal soup intake							
Less than monthly	1,070 (10.51)	7,026.34	23	327.34	1.00	1.00	
Monthly or more	4,140 (40.67)	26,093.20	23	88.15	0.30 (0.17, 0.54)	0.31 (0.16, 0.62)	0.001
Weekly or more	4,969 (48.82)	30,210.32	23	76.13	0.25 (0.14, 0.45)	0.31 (0.15, 0.62)	0.001
*p* for trend							0.008
Duration of herbal soup intake (years)							
≤5	626 (6.10)	4,052.80	24	592.18	1.00	1.00	
>5	9,553 (93.90)	59,277.06	45	75.91	0.15 (0.09, 0.24)	0.29 (0.16, 0.51)	<0.001
Herbal tea intake							
Never consumption	5,036 (49.47)	30,888.44	37	119.79	1.00	1.00	
Ever consumption	5,143 (50.53)	32,441.41	32	98.64	0.80 (0.49, 1.29)	1.32 (0.68, 2.55)	0.416
Frequency of herbal tea intake							
Less than monthly	5,036 (49.47)	30,888.44	37	119.79	1.00	1.00	
Monthly or more	3,667 (36.03)	22,973.50	25	108.82	0.88 (0.53, 1.47)	1.50 (0.75, 2.97)	0.249
Weekly or more	1,476 (14.50)	9,467.91	7	73.93	0.57 (0.25, 1.28)	0.91 (0.35, 2.32)	0.836
*p* for trend							0.996
Duration of herbal tea intake (years)							
≤5	4,675 (45.93)	28,702.78	32	111.49	1.00	1.00	
>5	5,504 (54.07)	34,627.08	37	106.85	0.88 (0.54, 1.42)	0.70 (0.37, 1.34)	0.286

a Per 100,000 person-years.

b Adjusting for sex and age in minimally adjusted models.

c Adjusting for sex, age, education level, family history of NPC, combined EBV antibodies, smoking status, fresh fruits, fresh vegetables, and salted food in the fully adjusted model.

Herbal soup consumption showed a significant decrease in NPC risk (fully adjusted HR: 0.31, 95% CI: 0.16–0.58). Compared with infrequent consumption, participants who consumed herbal soup more frequently was associated with decreased risk of NPC, with HRs of 0.30 (95% CI: 0.17–0.54) and 0.25 (95% CI: 0.14–0.45) for those who consumed monthly or more and weekly or more in minimal models. Following full adjustment, the association was slightly attenuated, with HRs of 0.31 (95% CI: 0.16–0.62) and 0.31 (95% CI: 0.15–0.62) for those who consumed monthly or more and weekly or more, respectively. Compared with those who consumed herbal soup ≤5 years, consuming duration of >5 years showed a significant decrease in risk of NPC (fully adjusted HR: 0.29, 95% CI: 0.16–0.51). The association trend showed a clear enhancement when frequency and duration of herbal soup consumption were estimated jointly, with the corresponding HRs ranging from 0.07 to 0.13 (**Table 3**). After stratified analyses by potential effect modifiers, the association of herbal soup consumption frequency or duration with NPC risk was consistent across participants, irrespective of sex, age, education level, combined levels of EBV antibodies, smoking status, and salted food intake (**Supplementary Table 1**).

**Table 3:** Joint association between herbal diet intake frequency/duration and nasopharyngeal carcinoma risk in a prospective cohort in Sihui, southern China (*N* = 10,179).

Frequency of herbal diet intake	Duration of herbal diet intake				*p* for trend
	≤5 years		>5 years		
	*n*	Adjusted HR (95% CI)^a^	*n*	Adjusted HR (95% CI)^a^	
Herbal soup intake					
Less than monthly	133	1.00	937	0.09 (0.03,0.28)	<0.001
Monthly or more	269	0.13 (0.04,0.42)	3,871	0.07 (0.03,0.14)	
Weekly or more	224	0.09 (0.02,0.39)	4,745	0.07 (0.03,0.14)	
Herbal tea intake					
Less than monthly	4,312	1.00	724	1.00 (0.41,2.45)	0.634
Monthly or more	248	2.42 (1.00,5.86)	3,419	0.98 (0.53,1.83)	
Weekly or more	115	0.62 (0.08,4.74)	1,361	0.74 (0.29,1.86)	

a Adjusting for sex, age, education level, family history of NPC, combined EBV antibodies, smoking status, fresh fruits, fresh vegetables, and salted food in the Cox regression models.

In addition, the positive associations for NPC with salted food intake, family history of NPC, and tobacco smoking were observed in current study, with the corresponding adjusted HRs of 2.05 (95% CI: 1.18–3.55), 2.94 (95% CI: 1.38–6.25), and 3.22 (95% CI: 1.46–7.11) in the fully adjusted model. A very strong association between EBV seropositivity and NPC risk was also observed, with fully adjusted HR of 5.44 (95% CI: 2.68–11.07). However, sex and education level appeared to have no relationship with NPC in this cohort.

### Herbal Diet and EBV Seropositivity

**Figure 2:**
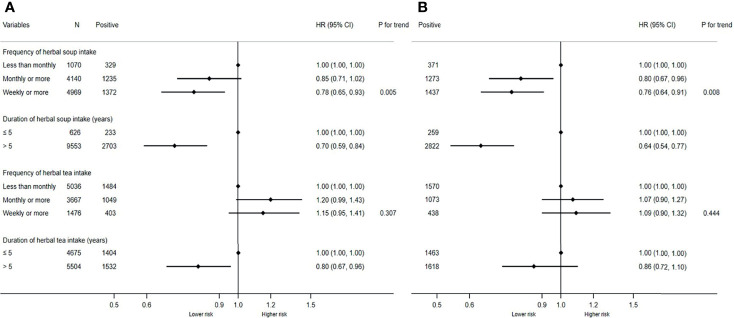
Associations of herbal diet with Epstein-Barr virus (EBV) seropositivity in all participants at baseline. **(A)** Associations of herbal diet with VCA-IgA seropositivity in all participants at baseline. **(B)** Associations of herbal diet with EBNA1-IgA seropositivity in all participants. The two logistic regression models were both adjusted for sex, age, education level, family history of NPC, smoking status, fresh fruits, fresh vegetables, and salted food. Positive, participants number with positive VCA-IgA or EBNA1-IgA.

Considering that the ingredients in herbal diet may mediate with EBV reactivation to affect NPC occurrence, we additionally explored the relationship between them among all the participants at baseline, as well as non-NPC individuals in this cohort. In all the participants, those with higher frequency or longer duration showed decreased seropositivity of the two EBV markers compared with those with less frequency and shorter duration (**Figure 2**). After adjusting for potential confounders, the adjusted ORs for VCA-IgA were respectively 0.78 (95% CI: 0.65–0.93) and 0.70 (95% CI: 0.59–0.84) in those with the highest frequency and with longer duration. Similarly, the adjusted ORs for EBNA1-IgA were 0.76 (95% CI: 0.64–0.91) and 0.64 (95% CI: 0.54–0.77) in the participants with these two categorizations. Moreover, we found statistically significant monotonic trends toward lower VCA-IgA (*p* for trend = 0.005) and EBNA1-IgA (*p* for trend = 0.008) seropositivity with increasing frequency of herbal soup consumption. Similar inverse associations were also observed in non-NPC individuals. The adjusted ORs for VCA-IgA were 0.78 (95% CI: 0.65–0.93) and 0.70 (95% CI: 0.58–0.83) in those with the highest frequency and with longer duration, respectively. Also, the adjusted ORs for EBNA1-IgA were 0.77 (95% CI: 0.65–0.92) and 0.66 (95% CI: 0.55–0.79) in the participants with these two categorizations (**Supplementary Figure 1**). However, we failed to find the association between herbal tea and EBV seropositivity.

## Discussion

### Principal Findings

In this large, prospective cohort study, we found that herbal soup consumption was associated with a decreased risk of NPC in southern China, but this association was not found with herbal tea. The inverse association with herbal soup consumption was not modified by any other NPC potential risk factors. The frequency and duration of herbal soup consumption were both inversely associated with NPC risks; moreover, this association became stronger when the two indictors were combined together. Our study is the first to examine the two kinds of herbal diet with EBV reactivation in a large population in NPC endemic area. We found that both herbal soup consumption frequency and duration were inversely associated with EBV seropositivity among all the participants at baseline, as well as the non-NPC individuals in this cohort. It confirms an anti-EBV biological pathway by which herbal soup could inhibit NPC development.

### Comparison With Other Studies and Potential Mechanisms

Traditional herbal teas in southern China contain anti-inflammatory ingredients (33) and are usually used to alleviate some symptoms associated with sticky and humid conditions in this region (24). The association between herbal tea and NPC risk has been evaluated in several case-control studies in southern China, but the results were inconsistent. One study in Guangxi, China reported that herbal tea consumption was associated with an elevated risk of NPC (34) and another study in Guangdong, China reported an inverse association (20). Whereas, the other two studies in southern China found no association with NPC risk (35). The reasons for the discrepancy might lie in the influence of reverse causality, confounding bias, and chance in case-control studies. The prospective design of our cohort study with long-term follow-up can overcome the shortcomings of retrospective study and show the real relationship of herbal tea with NPC development. The overall null finding suggests that anti-inflammatory treatment with herbal tea or other ways is unlikely to contribute to NPC prevention.

It is interesting to notice a moderate increase in the risk of NPC among those who consumed herbal tea monthly or more for ≤5 years in the combined analysis of duration and frequency of herbal tea consumption (**Table 3**). This result might be due to reverse causality, which is similar to our prior finding in a population-based case-control study (19). It is possible that the early symptoms of NPC may be mistaken for influenza-like symptoms (36, 37), and the patients may consume herbal tea more frequently during a short time to alleviate discomfort before a clinical diagnosis.

In addition to herbal teas, consumption of soups with complex herbal ingredients is common in local residents in southern China (13). In our study, the results showed that 40.67% of participants consumed herbal soup monthly or more and 48.82% weekly or more. Consistent with the results of two case-control studies in Guangdong, China (19, 20), our study found that intake of herbal soup is associated with decreased risk of NPC, and our results showed a stronger effect than previous studies. This may be due to the prospective design of the current study, which can diminish recall bias and reverse causality and illustrate the true degree of effectiveness. The strong association may be also driven by some unmeasured confounders, such as genetic background, occupational exposure, air pollution in domicile, history of chronic ear, nose, throat, or chronic respiratory tract conditions, social and economic levels, and so on. Although the association between frequency of herbal soup consumption and NPC was insignificant in several stratums after stratified analysis, it exhibited beneficial effect against NPC. A limited number of NPC cases in these subgroups may account for this.

Distinguished with herbal tea for the main purpose of being anti-inflammatory, people consume herbal soup with discrepant herbal ingredients to maintain energy and improve immunity. According to our previous study, nine commonly used herbal plants species (*Ziziphus jujuba*, *F. lycii*, *Codonopsis pilosula*, *Astragalus membranaceus*, *S. coicis*, *Smilax glabra*, *Pristimantis calcaratus*, *Morinda officinalis*, and *Atractylodes macrocephala*) in herbal soups might have been involved in the anticancer effect through inducing apoptosis, inhibiting cancer cell migration and differentiation, or regulating signaling pathway (19, 38–42). As many studies *in vitro* have shown that bioactive components in herbal soup may inhibit EBV (25, 43, 44), the most important risk factor for NPC (2), an association between herbal diet and NPC through antiviral biological mechanisms have often been hypothesized but never thoroughly investigated.

In the current study, we found both herbal soup consumption frequency and duration were inversely associated with EBV seropositivity among all participants at baseline, as well as non-NPC population in this cohort. Statistically significant monotonic trends toward lower EBV seropositivity with increasing frequency of herbal soup consumption were also observed. The evidence of mechanisms that herbal medicines inhibit EBV activation is limited but could partly be attributed to their antiviral and immune-enhancing effects (38, 45–49). We noticed several studies have revealed that some herbal medicines such as *Croton tiglium*, *Euphorbia kansui*, *Daphne genkwa*, *Wikstroemia chamaedaphne*, *Wikstroemia indica*, etc. can activate EBV or promote EBV-transformed cells (29, 50), but these herbs are often used for clinical treatment of special diseases rather than being used in herbal diet consumed in daily life.

### Strengths and Limitations

To our knowledge, this study is the first large-scale cohort study to evaluate the long-term NPC risk with traditional herbal diet in southern China. The detailed investigation of NPC risk with the indictors of the herbal diet consumption and stratified analysis with well-known NPC potential confounders strengthens our conclusion for the inverse association between herbal soup consumption and NPC. Furthermore, we tested EBV antibodies among all the participants, which enables us to confirm that the inverse association between herbal soup and NPC risk is partly mediated by EBV inactivation.

There are also several limitations in this study. First of all, our cohort was mainly conducted in southern China and the findings might not be generalizable to the nonendemic regions. Secondly, we only obtained the information of herbal diet consumption at baseline and did not obtain the changing status of this habit in the participants, which may affect the association magnitude. Considering that consumption of herbal diet is a traditional life habit, we assume this change would be minor. Third, although we carefully considered traditional risk factors in analyses, there might be effects of unmeasured confounding factors in our study. Further well-designed studies collecting more potential risk factors and in more regions would contribute to the rigorous evaluation of herbal soup for NPC risk.

## Conclusions

Overall, our study provided a prospectively promising evidence for a reduced NPC risk with the consumption of herbal soup. We also revealed that the inverse association is partly attributed to inhibition of EBV reactivation by herbal plants in herbal soups. Due to convenience and relative safety for long-term use, developing the habits of herbal soup consumption might be an effective way for NPC prevention in high-risk populations.

## Data Availability Statement

The original contributions presented in the study are included in the article/**Supplementary Material**. Further inquiries can be directed to the corresponding authors.

## Ethics Statement

The studies involving human participants were reviewed and approved by the Institutional Research Ethics Committee of Sun Yat-Sen University Cancer Center. The patients/participants provided their written informed consent to participate in this study.

## Author Contributions

Y-HL analyzed the data and wrote the manuscript. C-YL conducted questionnaire investigation, collected data, and did data cleaning. S-HX and TL contributed to data collection and database management. QL provided support on statistics. WL and Y-QL contributed to recruitment of study population or acquisition of data. S-MC and A-HL contributed to the conception and design of the work. All authors contributed to the article and approved the submitted version.

## Funding

This study was supported by the South China Cohort of Chronic Diseases (Award Number: 2017YFC0907102), the National Key Research and Development Program of China (Award Number: 2020YFC1316905), the National Natural Science Foundation of China (Award Number: 81872700, 8207362), and Planned Science and Technology Project of Guangdong Province (Award Number: 2017A020215033).

## Conflict of Interest

The authors declare that the research was conducted in the absence of any commercial or financial relationships that could be construed as a potential conflict of interest.

## Publisher’s Note

All claims expressed in this article are solely those of the authors and do not necessarily represent those of their affiliated organizations, or those of the publisher, the editors and the reviewers. Any product that may be evaluated in this article, or claim that may be made by its manufacturer, is not guaranteed or endorsed by the publisher.
