# Supplementary material for: Association Between Traditional Herbal Diet and Nasopharyngeal Carcinoma Risk: A Prospective Cohort Study in Southern China
Source: Front Oncol. 2021 Oct 21;11:715242. doi: 10.3389/fonc.2021.715242 (PMC8566915; doi:10.3389/fonc.2021.715242)
Supplement: Supplementary file 1 [file DataSheet_1.pdf]

Supplementary Material

1 Supplementary Tables

Supplementary Table 1. Associations between herbal soup consumption and nasopharyngeal carcinoma stratified by potential risk factors.

Variables [a]	Frequency of herbal soup intake [a]			*P*-value for interaction	Duration of herbal soup intake [a]		*P*-value for interaction
	Less than monthly	Monthly or more	Weekly or more		≤ 5 years	> 5 years	
Sex							
Male	1.00	0.24(0.11,0.57)	0.25(0.11,0.60)	0.907	1.00	0.38(0.18,0.81)	0.443
Female	1.00	0.57(0.15,2.12)	0.57(0.15,2.24)		1.00	0.16(0.06,0.40)	
Age (years)							
< 50	1.00	0.23(0.09,0.59)	0.23(0.08,0.61)	0.456	1.00	0.25(0.11,0.58)	0.880
≥ 50	1.00	0.43(0.16,0.19)	0.43(0.16,1.20)		1.00	0.28(0.13,0.64)	
Education year (years)							
< 6	1.00	0.46(0.16,1.54)	0.48(0.14,1.65)	0.877	1.00	0.35(0.16,0.90)	0.219
≥ 6	1.00	0.28(0.12,0.70)	0.28(0.11,0.68)		1.00	0.20(0.10,0.43)	
Combined EBV antibodies							
Both negative	1.00	0.18(0.03,1.16)	0.29(0.04,1.96)	0.649	1.00	0.12(0.02,0.57)	0.062
Any positive	1.00	0.34(0.16,0.72)	0.32(0.15,0.69)		1.00	0.29(0.16,0.55)	
Smoking status							
Never smoker	1.00	0.38(0.12,1.23)	0.27(0.08,0.93)	0.317	1.00	0.16(0.06,0.40)	0.306
Ever smoker	1.00	0.28(0.12,0.68)	0.35(0.15,0.85)		1.00	0.34(0.16,0.72)	
Salted food intake							
Less than monthly	1.00	0.20(0.06,0.72)	0.10(0.02,0.46)	0.320	1.00	0.23(0.06,0.86)	0.866
Monthly or more	1.00	0.36(0.15,0.86)	0.47(0.20,1.09)		1.00	0.24(0.12,0.48)	

[a] Stratified analyses were performed by the following variables: age, sex, education level, combined EBV antibodies, smoking status and salted food intake.

Supplementary Table 2. Associations between herbal tea consumption and nasopharyngeal carcinoma stratified by potential risk factors.

Variable	Frequency of herbal tea intake [a]			*P*-value for interaction	Duration of herbal tea intake [a]		*P*-value for interaction
	Less than monthly	Monthly or more	Weekly or more		≤ 5 years	> 5 years	
Sex							
Male	1.00	1.86(0.81,4.26)	1.63(0.55,4.88)	0.489	1.00	0.49(0.23,1.07)	0.314
Female	1.00	0.79(0.21,2.98)	0.19(0.02,1.83)		1.00	1.98(0.52,7.55)	
Age (years)							
< 50	1.00	1.43(0.54,3.79)	0.60(0.12,2.91)	0.188	1.00	0.54(0.21,1.38)	0.237
≥ 50	1.00	1.59(0.61,4.15)	1.14(0.33,3.94)		1.00	1.00(0.41,2.43)	
Education year (years)							
< 6	1.00	1.76(0.73,4.23)	1.25(0.39,4.00)	0.739	1.00	1.51(0.47,4.85)	0.223
≥ 6	1.00	1.09(0.33,3.60)	0.45(0.08,2.55)		1.00	0.60(0.25,1.32)	
Combined EBV antibodies							
Both negative	1.00	1.88(0.22,16.03)	0.55(0.02,12.72)	0.786	1.00	0.27(0.03,2.33)	0.175
Any positive	1.00	1.26(0.61,2.64)	0.73(0.26,2.03)		1.00	0.92(0.46,1.85)	
Smoking status							
Never smoker	1.00	1.17(0.35,3.88)	0.60(0.11,3.34)	0.916	1.00	0.55(0.25,1.22)	0.254
Ever smoker	1.00	1.57(0.67,3.66)	1.01(0.32,3.14)		1.00	1.37(0.41,4.60)	
Salted food intake							
Less than monthly	1.00	1.06(0.46,2.45)	0.72(0.24,2.12)	0.408	1.00	0.36(0.11,1.16)	0.053
Monthly or more	1.00	3.64(0.99,13.42)	0.99(0.11,8.92)		1.00	1.24(0.55,2.78)	

[a] Stratified analyses were performed by the following variables: age, sex, education level, combined EBV antibodies, smoking status and salted food.

2 Supplementary Figures

Supplementary Figure 1. Associations of herbal diet with Epstein-Barr virus (EBV) seropositivity in non-NPC individuals. (A) Associations of herbal diet with VCA-IgA seropositivity in non-NPC individuals. (B) Associations of herbal diet with EBNA1-IgA seropositivity in non-NPC individuals. The two logistic regression models were both adjusted for sex, age, education level, family history of NPC, smoking status, fresh fruits, fresh vegetables and salted food. Positive: Participants number with positive VCA-IgA or EBNA1-IgA.
