# Supplementary figures and images for: Association Between Traditional Herbal Diet and Nasopharyngeal Carcinoma Risk: A Prospective Cohort Study in Southern China
Source: Front Oncol. 2021 Oct 21;11:715242. doi: 10.3389/fonc.2021.715242 (PMC8566915; doi:10.3389/fonc.2021.715242)

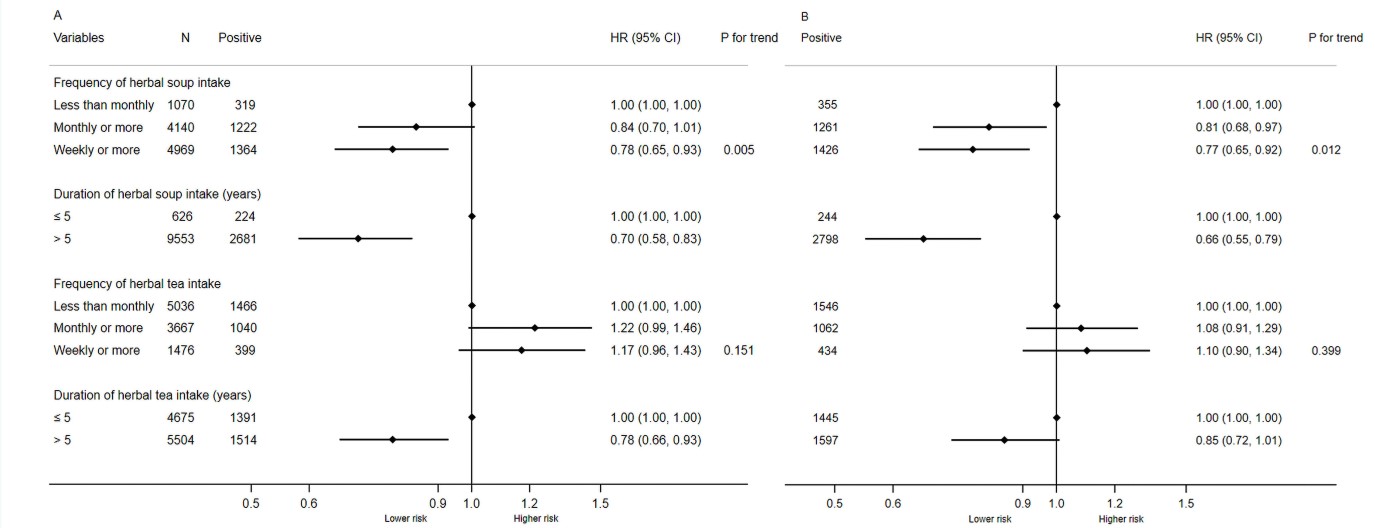

Supplement: Supplementary file 2 [file Image_1.jpeg]
